# Glutathione-S-Transferase: A Minor Allergen in Birch Pollen due to Limited Release from Hydrated Pollen

**DOI:** 10.1371/journal.pone.0109075

**Published:** 2014-10-02

**Authors:** Stephan Deifl, Christian Zwicker, Eva Vejvar, Claudia Kitzmüller, Gabriele Gadermaier, Birgit Nagl, Susanne Vrtala, Peter Briza, Gerhard J. Zlabinger, Beatrice Jahn-Schmid, Fatima Ferreira, Barbara Bohle

**Affiliations:** 1 Christian Doppler Laboratory for Immunomodulation, Medical University of Vienna, Vienna, Austria; 2 Department of Pathophysiology and Allergy Research, Medical University of Vienna, Vienna, Austria; 3 Christian Doppler Laboratory for Allergy Diagnosis and Therapy, University of Salzburg, Salzburg, Austria; 4 Department of Molecular Biology, University of Salzburg, Salzburg, Austria; 5 Institute of Immunology, Medical University of Vienna, Vienna, Austria; Université Paris Descartes, France

## Abstract

**Background:**

Recently, a protein homologous to glutathione-S-transferases (GST) was detected in prominent amounts in birch pollen by proteomic profiling. As members of the GST family are relevant allergens in mites, cockroach and fungi we investigated the allergenic relevance of GST from birch (bGST).

**Methodology:**

bGST was expressed in *Escherichia coli*, purified and characterized by mass spectrometry. Sera from 217 birch pollen-allergic patients were tested for IgE-reactivity to bGST by ELISA. The mediator-releasing activity of bGST was analysed with IgE-loaded rat basophil leukaemia cells (RBL) expressing human FcεRI. BALB/c mice were immunized with bGST or Bet v 1. Antibody and T cell responses to either protein were assessed. IgE-cross-reactivity between bGST with GST from house dust mite, Der p 8, was studied with murine and human sera in ELISA. The release kinetics of bGST and Bet v 1 from birch pollen were assessed in water, simulated lung fluid, 0.9% NaCl and PBS. Eluted proteins were quantified by ELISA and analysed by immunoblotting.

**Principle findings:**

Only 13% of 217 birch pollen-allergic patients showed IgE-reactivity to bGST. In RBL assays bGST induced mediator release. Immunization of mice with bGST induced specific IgE and a Th2-dominated cellular immune response comparably to immunization with Bet v 1. bGST did not cross-react with Der p 8. In contrast to Bet v 1, only low amounts of bGST were released from pollen grains upon incubation in water and the different physiological solutions.

**Conclusion/Significance:**

Although bGST is abundant in birch pollen, immunogenic in mice and able to induce mediator release from effector cells passively loaded with specific IgE, it is a minor allergen for birch pollen-allergic patients. We refer this discrepancy to its limited release from hydrated pollen. Hence, bGST is an example demonstrating that allergenicity depends mainly on rapid elution from airborne particles.

## Introduction

Pollen from birch (*Betula verrucosa*) represents an important source of inhalant allergens in Central and Northern Europe, North America, and Japan [1]. The single major allergen Bet v 1 belongs to the family of pathogenesis-related (PR)-10 proteins and displays a characteristic structure termed the Bet v 1-fold [2]. So far, five additional allergens have been identified in birch pollen (BP) which are all minor allergens. Bet v 2 belongs to the family of profilins [3], [4]. Bet v 3 and Bet v 4 are Ca^2+^-binding proteins [5], [6]. Bet v 6 was identified as an isoflavone reductase-related protein [7] and Bet v 7 was reported to be a member of the cyclophilin family. All allergens in BP show high homology with proteins in other pollen species or plant foods which causes vast immunological cross-reactivity [3], [8]–[11].

Recently, the protein profile of BP was analyzed by mass spectrometry [12]. In this study a 27 kDa protein belonging to the glutathione-S-transferase (GST) family was identified. GST represent a well-conserved and multifunctional enzyme superfamily and are present in virtually all organisms from bacteria to man [13]. These enzymes catalyze the detoxification of reactive endogenous compounds or xenobiotics by conjugation of glutathione to mainly electrophilic substrates. GST-homologues in house dust mite (HDM, *Dermatophagoides pteronyssinus*, Der p 8) and German cockroach (*Blattella germanica*, Bla g 5) have been identified as relevant allergens. For example, 40–96% of HDM-allergic patients have been shown to possess IgE-responses to Der p 8 [14], [15] and 30–70% of cockroach-allergic individuals display specific IgE for Bla g 5 [16]–[19]. Another GST, Alt a 13, has been identified as major allergen in *Alternaria alternata* [20]. IgE-cross-reactivity between allergenic GST from mites, cockroach, fungi and helminths has been reported [15], [21]–[23]. Recently, GST in wheat (*Triticum aestivum*) has also been identified as an allergen [24].

The aim of this study was to explore the allergenic relevance of GST in BP (bGST). The protein was cloned from BP cDNA and expressed in *E.coli*. Antibody (Ab) and T cell responses to bGST were evaluated in mice and BP-allergic patients. The cross-reactivity between bGST and Der p 8 was analyzed. Finally, the release kinetics of bGST from pollen grains in water and different physiologic solutions were compared to Bet v 1.

## Materials and Methods

### Recombinant bGST

Total RNA was extracted from BP with NucleoSpin RNA II Kit (Machery-Nagel, Dueren, Germany) and total cDNA was obtained with SuperScript III First-Strand Synthesis System for RT-PCR (Invitrogen, Carlsbad, CA, USA) using oligo(dT)-nucleotides. On the basis of the partial amino acid (aa) sequence that was retrieved from mass spectrometry [12], the DNA sequence was obtained by PCR using a degenerated 5′ CCNGCNTGGTAYAARGARAA 3′ forward and an oligo(dT)-nucleotide reverse primer. The missing 5′ end was obtained by subsequent RACE-protocols (FirstChoice RLM-RACE Kit, Ambion, Foster City, CA, USA) with a gene specific 5′ GTGATATCATACTTCAACGCGTCC 3′ reverse primer. A full length-clone was retrieved using 5′ GAGACATATGGCTGATGCGAGCGTGA 3′ forward and 5′GAGACTCGAGCTGCTGAGCCGC 3′ reverse primer with flanking NdeI and XhoI restriction sites for cloning into pHIS parallel2 expression vector [25]. bGST was expressed as a soluble, C-terminally His-tagged protein in *E. coli* BL21 DE3 cells and purified by Ni-affinity chromatography. The purity and identity of bGST was analyzed by SDS-PAGE and mass spectrometry as described [12]. The protein was applied to endotoxin removal columns (Endotrap Red, Hyglos, Bernried, Germany) and the final endotoxin concentration was 7 ng endotoxin/mg protein as determined by Limulus amebocyte lysate assay (Lonza, Basel, Switzerland). Circular dichroism spectra were recorded from 190 to 260 nm at 20°C and 95°C using a JASCO J-815 spectropolarimeter (Jasco, Tokyo, Japan). For determination of the melting point spectra were taken at 222 nm during thermal denaturation (20–95°C, ΔT = 1°C min^−1^).

### Allergens

Recombinant Bet v 1 and Bet v 2 were purchased from Biomay AG (Vienna, Austria). Recombinant Bet v 3, Bet v 4, Bet v 6, Bet v 7, and house dust mite extract (HDME) were produced as described [5]–[7]. Endotoxin levels of all allergens were ≤16 ng endotoxin/mg protein. To produce BP extract (BPE) commercially available BP (Allergon, Ängelholm, Sweden) was incubated in PBS for 6 h at RT and centrifuged for 30 min at 20000 rpm. The supernatant was filtered through a sterile filter (0.22 µm, Sartorius Stedim, Göttingen, Germany) and lyophilized.

### Assessment of human IgE

ELISA plates were coated with bGST (2 µg/mL), recombinant allergens (1 µg/mL), BPE or HDME (50 µg/mL). To assess allergen-specific IgE, sera from 217 BP-allergic individuals were tested in duplicates diluted 1∶4 and 1∶8. This study population has been described elsewhere [11]. Briefly, all patients suffered from rhinoconjunctivitis in spring and showed positive skin prick test responses to BPE and specific IgE to BP (≥0.35 kU_A_/L, ImmunoCAP, Thermofisher, Uppsala, Sweden). The sera were collected for the routine diagnosis of allergy and all individuals gave written informed consent that their samples can be further analysed for IgE-reactivity to BP proteins. None of the authors had participated in the collection of these sera, nor had interacted with donors or had access to any identifying information associated with the samples. Sera from eight Austrian non-allergic individuals served as negative controls. These samples were analyzed in an anonymous manner after informed written consent was obtained from the healthy individuals with approval of the local ethics committee, Medical University of Vienna, Austria (EK number 028/2006). The authors participated in the collection of the control samples. IgE was detected by alkaline-phosphatase conjugated anti-human IgE Ab (BD Pharmingen). The mean value of all non-allergic controls plus 5 times the standard deviation was set as cut-off for positive IgE-reactivity. For inhibition experiments sera were pre-incubated with the indicated concentrations of either bGST or BPE. Sera from HDM-allergic Der p 8-sensitized patients derived from a cohort described previously [26].

### Rat basophilic leukemia (RBL) cell mediator release

RBL assays were performed as previously described [27]. Briefly, FcεRI-humanized RBL-2H3 cells were passively sensitized with serum IgE from BP-allergic patients. After addition of serial dilutions of the respective proteins β-hexosaminidase was measured and expressed as percentage of the release obtained from anti-IgE-treated cells.

### Immunization protocol

Female BALB/c mice (6 weeks old, Charles River, Sulzfeld, Germany) were immunized intraperitoneally with either bGST or Bet v 1 in PBS adsorbed to Alum (100 µL, Serva GmbH, Heidelberg, Germany). Prior to each immunization blood was collected by tail bleeding. The animals received 10 µg of protein in 150 µL total volume on day 0, followed by 5 µg of protein/animal on day 14, 28, 35 and 49. On day 59 mice were sacrificed and spleens were removed under aseptic conditions. All experiments were approved by the Animal Experimentation Ethics Committee of the Medical University of Vienna and the Ministry of Science and Research (Permit number: GZ 66.009/0215-II/3b/2010). All efforts were made to minimize suffering.

### Antibody responses in BALB/c mice

ELISA plates (Nunc Maxisorp, Thermo Fisher Scientific, Waltham, MA, USA) were coated with bGST (2 µg/mL), Bet v 1 (1 µg/mL), BPE or HDME (each 50 µg/mL) in carbonate buffer (pH = 9.6). After saturation, sera were incubated overnight. For detection of IgG1 and IgG2a, sera were diluted 1∶1000; for IgG3 1∶400 and for IgE 1∶20. Rat anti-mouse IgG1, IgG2a, IgG3 and IgE (BD Pharmingen, San Jose, CA, USA) and a HRP-conjugated goat anti-rat IgG (GE Healthcare, Vienna, Austria) were used for detection. The absorbance was measured at 405 nm. For inhibition experiments, sera of bGST-immunized mice were diluted 1∶1000 and pre-incubated with different concentrations of bGST and BPE overnight at 4°C. Bovine serum albumin served as a negative control.

### Proliferative and cytokine responses of splenocytes

Splenocytes were isolated and incubated (2×10^5^/well) in round-bottom 96 well plates (Nunc) with bGST (0.39–2.5 µg/mL), Bet v 1 (5, 10 µg/mL), or medium alone for 4 days at 37°C. Concanavalin A (2.5 µg/mL; Sigma Aldrich, USA) served as positive control. Subsequently, ^3^[H]-labeled thymidine (0.5 µCi/mL) was added for 16 hours. To analyze cytokine responses, splenocytes (5×10^6^ cells) were left in medium alone or stimulated with bGST (1.25 µg/mL) or Bet v 1 (5 µg/mL) for 40 h. IL-2, IL-4, IL-5, IL-10, IL-13, IL-17 and IFN-γ levels in supernatants were measured by Multiplex cytokine assays (Luminex, Austin, TX, USA).

### Release of bGST and Bet v 1 from birch pollen grains

BP (50 mg/mL, Allergon) was incubated in tab water, 0.9% NaCl, PBS or simulated lung fluid. The latter was prepared according to Marques *et al* [28]. Briefly, MgCl_2_ (2.14 mM), NaCl (103 mM), KCl (4 mM), Na_2_SO_4_ (0.05 mM), CaCl_2_.2 H_2_O (2.5 mM), CH_3_COONa.3H_2_O (7 mM), NaHCO_3_ (31 mM), HOC(COONa)(CH_2_COONa)_2_.2H_2_O (0.33 mM) and NaH_2_PO_4_.H_2_O (1.03 mM) were solved in the given order in ddH_2_O and the pH was adjusted to 7.4. Samples were taken at indicated time points and centrifuged twice for 1 minute at 10000 rpm. Supernatants were stored at −20°C. Thawed supernatants (diluted 1∶10 and 1∶100, respectively) were used for coating of ELISA plates (Nunc Maxisorp, Nunc) in carbonate buffer (pH = 9.6). Bet v 1 or bGST were coated in different concentrations to generate a standard curve. Detection of bGST or Bet v 1 was done using the sera of bGST-immunized mice and a monoclonal anti-Bet v 1 antibody, respectively. A goat anti-mouse HRP-labelled antibody (GE Healthcare, Vienna, Austria) served as secondary antibody. Bovine serum albumin served as a negative control.

Thawed supernatants were separated by SDS-PAGE (15%) under non-reducing conditions. After blotting onto a nitrocellulose membrane, proteins were detected using sera from mice immunized with bGST or Bet v 1 and a goat anti-mouse HRP-labelled Ab (GE Healthcare). ECL prime detection reagent (GE Healthcare) was used for luminescence. This experiment was performed twice.

## Results

### Characterization of recombinant bGST

**Figure 1:**
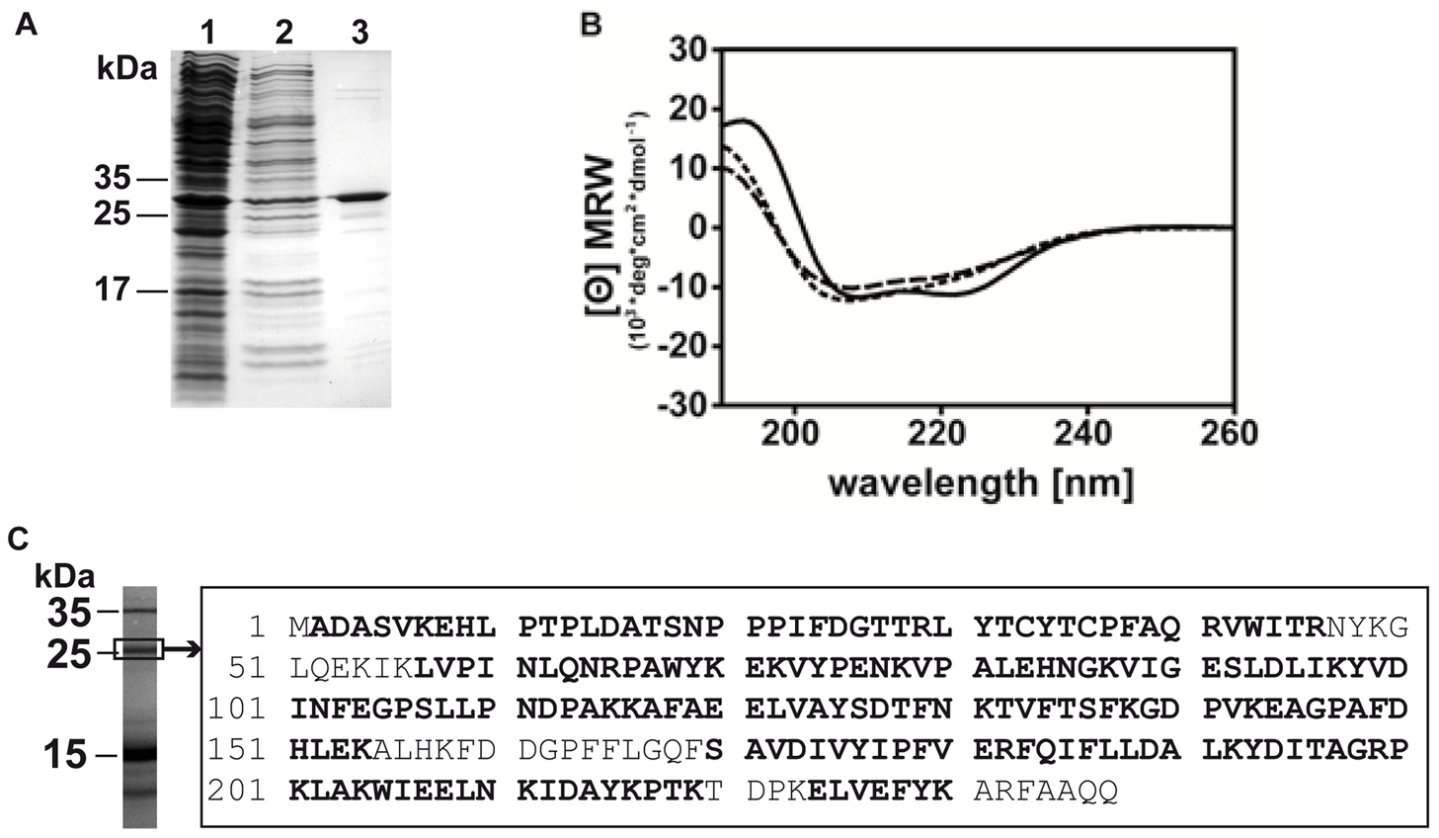
Purity and identity of bGST. (A) Coomassie-stained SDS-PAGE showing bGST before (lanes 1 and 2) and after Ni^2+^ affinity-chromatography (lane 3). (B) Circular dichroism spectroscopy of bGST. Far UV-measurements of bGST were recorded at 20°C (solid line), 95°C (dashed line) and 20°C after renaturation (dotted line). The mean residue ellipticities (⊖) are shown at given wavelengths. (C) Natural bGST was excised from a Coomassie-stained SDS-PAGE of BPE and analysed by mass spectrometry. Bold characters show aa residues of natural bGST in the sequence of recombinant bGST.

The full-length cDNA sequence of bGST (NCBI accession number KF246508) was obtained using a degenerated sequence-specific forward primer in combination with an oligo(dT) nucleotide, followed by 5′RACE. The mature sequence of bGST is represented by 237 amino acids and has a theoretical molecular weight of 27,039.0 Da and a pI of 6.45. Recombinant bGST was expressed and purified to homogeneity (Fig. 1A). To confirm the validity of the cloned sequence, birch pollen extract was separated by SDS-PAGE, the band corresponding to natural GST was excised and in-gel digested with trypsin. The resulting peptides were analyzed by LC-MS/MS. Coverage was 85%, thus corroborating the sequence obtained by cloning (Fig. 1C). Analysis of the natural bGST also showed that the start methionine was missing and that the N-terminal alanine was acetylated. Circular dichroism spectroscopy of bGST revealed curves with a maximum at 193 nm and double minima at 209 and 222 nm, respectively, indicating the presence of a properly folded protein (Fig. 1B). A melting point of 72°C was obtained. Sequence comparison with GST from various sources revealed that bGST is most homologous to GST from *Jatropha curcas* (purge nut) displaying 79% sequence identity and to GST from *Ricinus communis* displaying 77% sequence identity. These GST belong to the omega subclass.

### Prevalence of human IgE to bGST and known BP allergens

**Figure 2:**
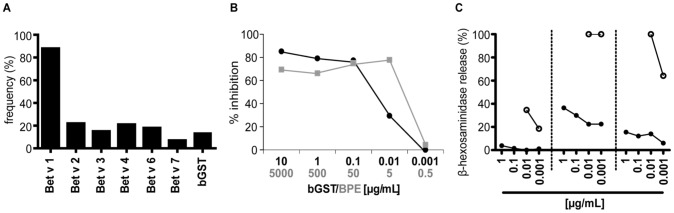
IgE-responses of BP-allergic individuals. (A) IgE-reactivity of 217 BP-allergic patients to BP allergens and bGST. (B) IgE-reactivity to bGST is inhibited by pre-incubation of sera with BPE (gray line) and bGST (black line). One representative example of four is shown. (C) RBL assay with sera from three patients sensitized to bGST and Bet v 1. Degranulation was induced with titrated amounts of bGST (filled circles) and Bet v 1 (open circles). The release of β-hexosaminidase is expressed as percentage of the release from anti-IgE-treated cells.

Sera obtained from 217 BP-allergic patients [11] were screened for IgE-reactivity to bGST and the known allergens in BP. 88% of the patients showed IgE-reactivity to Bet v 1, 22% to Bet v 2, 15% to Bet v 3, 21% to Bet v 4, 18% to Bet v 6 and 7% IgE to Bet v 7 (Fig. 2A). 29/217 (13.4%) patients displayed IgE specific for bGST. To investigate whether recombinant bGST contains the IgE epitopes of its natural counterpart, sera of 4 GST-sensitized individuals were pre-incubated with either bGST or BPE and then incubated with solid phase-bound bGST. BPE showed a comparable capacity to inhibit IgE-binding as the recombinant protein (Fig. 2B). Next, RBL assays were performed with sera from three patients showing IgE-reactivity to bGST (OD_405_>1.0) and Bet v 1 (Fig. 2C). Both recombinant proteins induced mediator release demonstrating allergenic activity.

### Murine immune responses to bGST and Bet v 1

**Figure 3:**
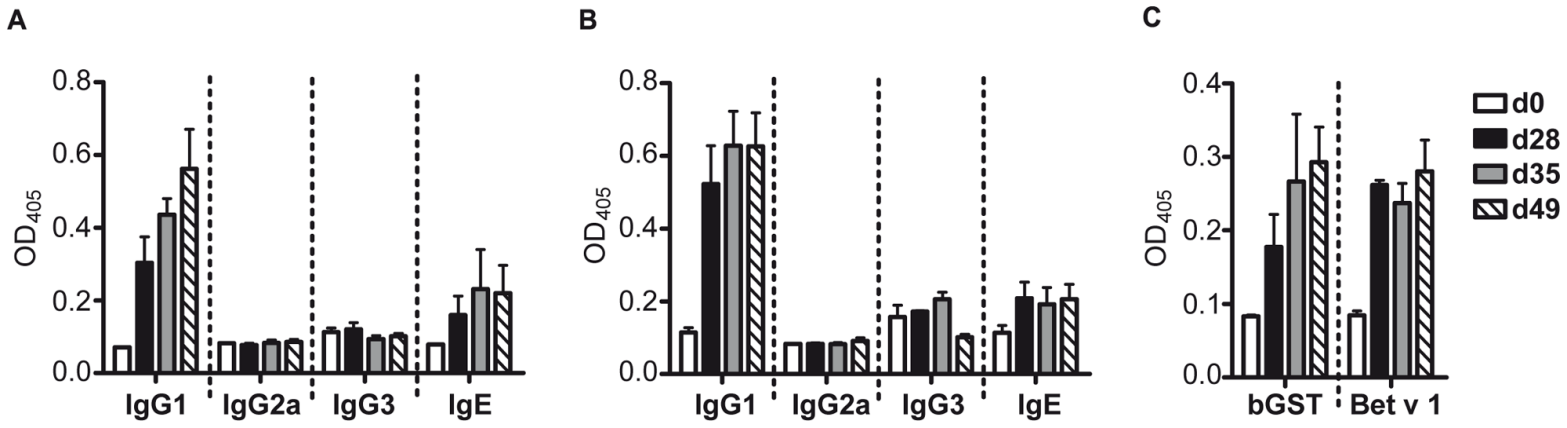
Ab responses to bGST and Bet v 1 in BALB/c mice. (A) bGST-specific Ig of mice immunized with bGST (n = 5). (B) Bet v 1-specific Ig of mice immunized with Bet v 1 (n = 5). (C) IgG1 responses to BPE from mice immunized with either bGST or Bet v 1. O.D.; optical density, d, day; mean values+standard deviation are shown.

**Figure 4:**
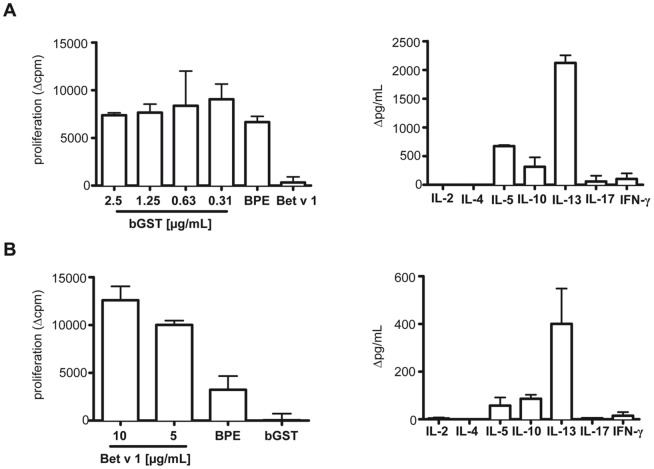
T cell responses to bGST and Bet v 1 in BALB/c mice. (A) Proliferative and cytokine responses of splenocytes from mice immunized with bGST (n = 5) to titrated amounts of bGST, BPE (25 µg/mL) and Bet v 1 (5 µg/mL). Cytokines were induced with 1.25 µg/mL of bGST. (B) Proliferative and cytokine responses of splenocytes from mice immunized with Bet v 1 (n = 5) to Bet v 1, BPE (25 µg/mL) and bGST (1.25 µg/mL). Cytokines were induced with 5 µg/mL of Bet v 1. Δcpm, cpm of unstimulated cultures were subtracted from stimulated cultures (mean background cpm = 4724±742); Δpg/mL, levels in unstimulated cultures were subtracted from stimulated cultures (mean pg/mL of IFN-γ were <135 pg/ml and of remaining cytokines <20 pg/ml).

BALB/c mice immunized with bGST produced specific IgG1 and IgE Abs but no detectable IgG2a and IgG3 (Fig. 3A). A similar Ab profile was observed after immunization with Bet v 1 (Fig. 3B). Murine Abs induced in response to immunization with either recombinant protein reacted with BPE indicating that they recognized their natural counterparts (Fig. 3C). To assess the T cell reactivity to either protein, splenocytes were stimulated with bGST, Bet v 1 and BPE, respectively. Splenocytes isolated from bGST-immunized mice proliferated upon stimulation with bGST and BPE but not with Bet v 1 (Fig. 4A). Splenocytes from Bet v 1-immunized mice proliferated in response to Bet v 1 and BPE but not to bGST (Fig. 4B). The T cell response to both proteins was characterized by the production of considerable amounts of IL-5, IL-13 and IL-10 and marginal levels of IFN-γ and IL-17 (Fig. 4A, B). IL-2 and IL-4 were not detected in response to either protein.

### Cross-reactivity of bGST and Der p 8

**Figure 5:**
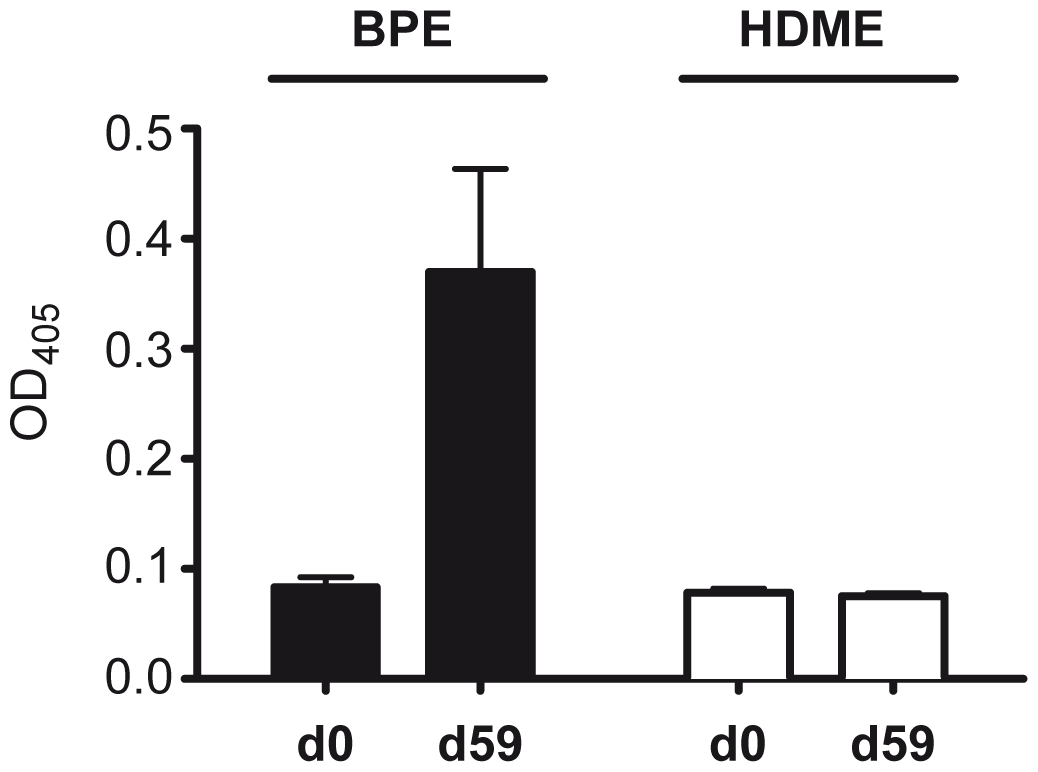
No cross-reactivity between bGST and HDM-GST. (A) IgG1-reactivity of sera (n = 3) collected from bGST-immunized mice at day 0 and day 59 to BPE and HDME. (B) IgE-reactivity of seven Der p 8-sensitized HDM-allergic patients to bGST and HDME. NHS, non-allergic control sera; O.D. optical density; dotted and dashed lines indicate the cut-off for positive IgE-reactivity.

The sera obtained from bGST-immunized mice did not react with HDME but were clearly positive with BPE (Fig. 5A). In addition, seven Der p 8-sensitized HDM-allergic patients were tested for their IgE-reactivity with bGST. Whereas all patients showed IgE-reactivity to HDME, none of the individuals reacted with bGST (Fig. 5B). Together, neither murine nor human sera indicated that bGST cross-reacted with Der p 8.

### Release kinetics of bGST and Bet v 1 from hydrated birch pollen

**Figure 6:**
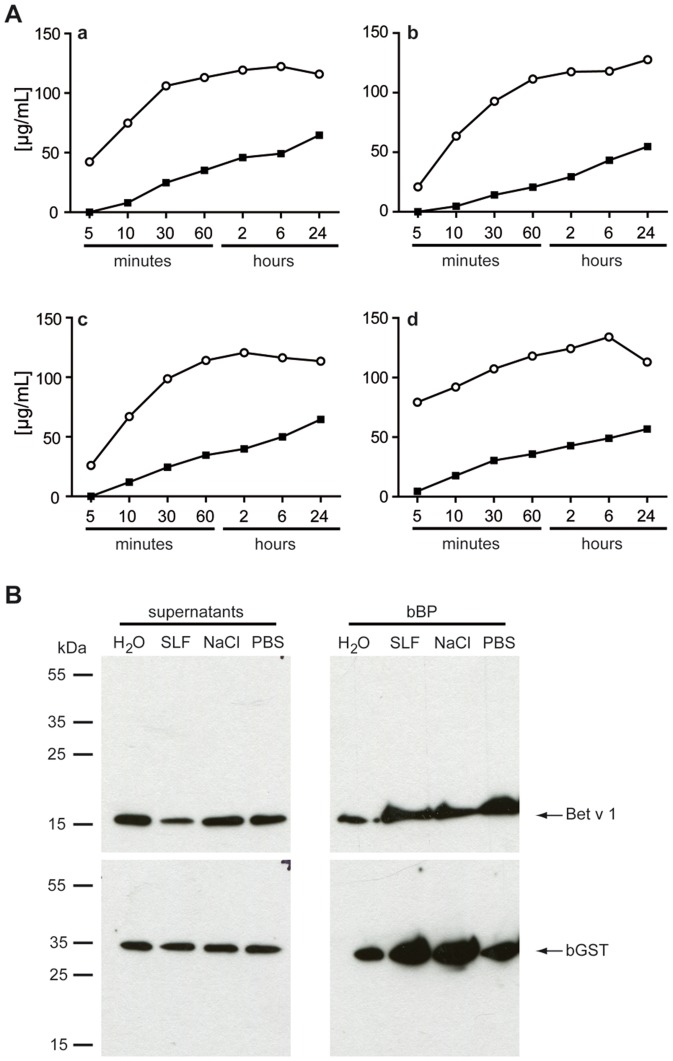
Release characteristics of bGST and Bet v 1 from BP. (A) Pollen grains were incubated in H_2_O (a), simulated lung fluid (b), 0.9% NaCl (c) and PBS (d). Supernatants taken at indicated time points were checked for their content of bGST (closed squares) and Bet v 1 (open circles) by ELISA. (B) Immunoblotting of supernatants collected after 60 min. bBP, boiled BP, SLF, simulated lung fluid.

Since allergenic properties of proteins have been linked to rapid solubility from airborne particles we assessed the release kinetics of bGST and Bet v 1 from BP grains upon contact with either water, simulated lung fluid, 0.9% NaCl or PBS within 24 hours. Matching previous data, considerable quantities of Bet v 1 were already detectable in all aqueous supernatants after 5 minutes of elution (Fig. 6A) [29]. The amounts of eluted Bet v 1 increased continuously and reached a plateau after about 60 minutes. In contrast, marginal amounts of eluted bGST were first detectable after 10 minutes of incubation of BP in the different solutions. The eluted amounts continuously increased over time. However, on average the amount of eluted bGST was approximately 4 times less than that of Bet v 1. To assess whether the formation of polymers or binding of bGST to other proteins prevented its release from pollen the supernatants collected after 60 minutes of incubation with the different solutions were separated under non-denaturing conditions. No aggregates were detected in immunoblots (Fig. 6B). Pollen grains disrupted by boiling in non-denaturing sample buffer served as positive control and confirmed that substantial amounts of both bGST and Bet v 1 were present in BP as single proteins.

## Discussion

Proteomic profiling has revealed that BP contains substantial amounts of a 27 kDa protein belonging to the GST family [12]. Since other members of this enzyme family represent highly allergenic proteins in various allergen sources we sought to investigate the relevance of bGST as an allergen in more detail. Since we did not succeed in purifying sufficient amounts of natural protein from BP, bGST was produced as soluble protein in *E. coli*. Mice were immunized with the purified recombinant protein to raise specific Ab. These Ab recognized natural GST in BP. Moreover, the binding of murine Abs and of human IgE to the recombinant protein was completely inhibited by BPE demonstrating that the recombinant protein contained all relevant epitopes of its natural counterpart. *Vice versa*, pre-incubation of murine sera with bGST blocked Ab-binding to BPE (data not shown). Together, these findings indicated the correct folding of recombinant bGST and its applicability as surrogate for the natural protein.

Bet v 1 is the major allergen in BP recognized by IgE from around 90% of BP-allergic individuals. Immunization of BALB/c mice with this protein adsorbed to alum typically induces Th2-polarized responses and specific IgE [30]. Immunization with bGST under the same conditions also promoted Th2-like responses and specific IgE production. Moreover, the protein was active in mediator release assays with FcεRI-humanized RBL cells passively loaded with IgE from bGST-sensitized patients. Thus, bGST might bear allergenic properties similar to Bet v 1. However, up to now bGST has not attracted attention as relevant allergen for BP-allergic patients. We confirmed a low prevalence of sensitization to bGST in a large patient cohort. Only 13% of 217 BP-allergic patients showed IgE-reactivity to bGST. The IgE-reactivity to other BP minor allergens ranged from 7–22%. Hence, bGST is among the least relevant allergens contained in BP. However, in contrast to other minor allergens, e.g. Bet v 2, Bet v 4 and Bet v 6, bGST is abundant in BP [12].

Several minor allergens in BP are panallergens cross-reacting with orthologs in various allergen sources. Cross-reactivity of GST from different mite species [15], [31] and fungal sources [22], as well as of GST from mites and cockroach [21] and recently also from cockroach and helminths has been demonstrated [23]. Therefore, we evaluated the possible cross-reactivity of bGST with HDM GST Der p 8, a prominent cross-reactive allergen [14], [26]. Neither bGST-specific murine Abs nor sera from Der p 8-sensitized patients reacted with Der p 8. The sequence homology between bGST and Der p 8 is 24%. bGST shares 22% homology with Bla g 5 and 32% with Alt a 13. Based on these low homologies we assume that bGST does not cross-react with these GST orthologs and therefore does not function as panallergen. Recently the aa sequence of a GST-like protein present in peach has been submitted to the protein data base (accession no. M5WH15). The aa sequence of this protein is 78% identical to bGST. Preliminary data in our laboratory indicated that the sera of bGST-immunized mice recognized GST in peach extract. We are now testing whether human bGST-specific IgE Ab also cross-react with the GST protein in peach.

Although bGST is abundant in BP, immunogenic in mice and capable of inducing mediator release from effector cells passively loaded with specific IgE, it is a minor allergen for BP-allergic patients. To investigate possible reasons for this discrepancy we assessed the release kinetics of bGST from hydrated pollen. Rapid elution from allergen sources on moisturized epithelial surfaces has been considered to contribute to the allergenicity of a protein. For example, immediate release from pollen has been reported for the major allergens Bet v 1, Ole e 1 from olive pollen, Phl p 1 and Phl p 5 from timothy grass pollen as well as for Der p 1 from fecal particles of HDM [29], [32], [33]. After their release, the passage of proteins through the mucosa in the respiratory tract and subsequent uptake and presentation by antigen-presenting cells are relevant first steps in the sensitization process. Comparing the release kinetics of bGST and Bet v 1 from pollen grains upon contact with water and different physiologic solutions concordantly revealed that bGST was eluted in much lower quantities than Bet v 1. Only marginal amounts of bGST were detected after 10 minutes of incubation in simulated lung fluid [28]. Based on the estimation that pollen grains remain in the nose for 6–8 minutes following inhalation [34], we speculate that the amount of bGST released from BP during this time period is too low to induce allergic sensitization.

The question “what makes a protein allergenic” is still not sufficiently answered. Several intrinsic features of proteins have been associated with allergenicity, for example enzymatic activity, lipid binding and activation of toll-like receptors, or glycosylation and binding to C-type lectins [35]. bGST is an example for an inhaled protein whose allergenicity depends mainly on the amount and speed of elution from airborne particles. In line with previous investigations, the present study stresses that rapid solubility from airborne particles is crucial for the initiation of the sensitization process.
